# Disparity-filtered differential correlation network analysis: a case study on CRC metabolomics

**DOI:** 10.1515/jib-2021-0030

**Published:** 2021-11-19

**Authors:** Silvia Sabatini, Amalia Gastaldelli

**Affiliations:** Institute of Clinical Physiology, CNR-Pisa, Via Moruzzi 1, Pisa, Italy; University of Siena, Siena, Italy

**Keywords:** colorectal cancer (CRC), differential network analysis, disparity filter, metabolomics

## Abstract

Differential network analysis has become a widely used technique to investigate changes of interactions among different conditions. Although the relationship between observed interactions and biochemical mechanisms is hard to establish, differential network analysis can provide useful insights about dysregulated pathways and candidate biomarkers. The available methods to detect differential interactions are heterogeneous and often rely on assumptions that are unrealistic in many applications. To address these issues, we develop a novel method for differential network analysis, using the so-called disparity filter as network reduction technique. In addition, we propose a classification model based on the inferred network interactions. The main novelty of this work lies in its ability to preserve connections that are statistically significant with respect to a null model without favouring any resolution scale, as a hard threshold would do, and without Gaussian assumptions. The method was tested using a published metabolomic dataset on colorectal cancer (CRC). Detected hub metabolites were consistent with recent literature and the classifier was able to distinguish CRC from polyp and healthy subjects with great accuracy. In conclusion, the proposed method provides a new simple and effective framework for the identification of differential interaction patterns and improves the biological interpretation of metabolomics data.

## Abbreviations

AUROC: area under the receiver operating characteristics
CRC: colorectal cancer
CV: cross-validation
FDR: false discovery rate
FNR: false negative rate
LC-MS/MS: liquid chromatography coupled with tandem mass spectrometry
NPV: negative predictive value
PLS-DA: partial least square-discriminant analysis
PPV: positive predictive value
VIP: variable importance in projection

## 1 Introduction

It is widely known that a complex biological phenomenon, such as the outbreak and the progression of cancer, is often the result of a complex web of interactions between several biological components, occurring at different levels. Recent advancements in ‘omics’ science allow to collect simultaneously large amounts of highly inter-related data, like metabolites, proteins, RNA, which may serve as good indicators for the status of a biological system in response to genetic, epigenetic, and environmental factors. This system of interactions finds a natural representation as a network: the biochemical molecules (e.g., metabolites or genes) may be seen as nodes and their pairwise relationships may be described as edges or links [1]. In metabolomics, the association between two molecules is frequently assessed by means of Pearson’s or Spearman’s correlation measures. In general, observed correlations are not sufficient to explain the underlying biochemical mechanisms, but it seems reasonable that, observing two phenotypes like disease and healthy status, differences or similarities in correlations can hint at altered or similar functional areas of the biological network [2–4], forming the basis for further analysis. This is the rationale for the use of differential network analysis, which aims to compare differential association patterns among different conditions. In this paper, we propose a novel procedure to conduct differential network analysis for metabolomics data, even if the workflow can be easily generalized to other types of data.

Although network approaches have been firstly adopted in transcriptomics or proteomics data, which are still the most frequently used in these fields, network analysis has proven to be particularly useful also in metabolomics, unveiling metabolic connections otherwise impossible to be detected relying solely on metabolites’ levels [5]. In particular, in most cases the greatest interest is in differential network analysis across different conditions [6]. In the last decades, several methods have been proposed to study differential network [3], mainly following two different approaches. The first consists in building one association network for each biological status and then comparing several aspects of networks topology [5, 7], [8], [9], like nodes’ strength and centrality. This approach is based on the assumption that nodes with greater changes in their connections are the ones which play a major role in the distinction of the two phenotypes. The second method, instead of defining separate association networks that require separate decisions and thresholds, consists in testing directly for differential associations and then building an edge-difference based differential network [10–12]. According to this approach, the first step is to define a dissimilarity measure to evaluate each pairwise differential interaction among the nodes between the two conditions. Besides correlation, the strength of such differential co-expressions has been also determined with measures based on mutual information [5], or partial correlation [13]. Then, a crucial step for the construction of the differential network is the identification of relevant edges to be included in the network. To accomplish this, the most common strategy is hard-thresholding [14], consisting in fixing an arbitrary cut-off to select meaningful interactions. Very few works have proposed alternatives to avoid such arbitrary choice: in [10] the authors used a permutation test to assess the statistical significance of each differential edges with respect to a null model, in [15] a soft-thresholding strategy for weighted graphs was proposed. Other techniques have been reviewed in [6, 16].

However, all presented methods face up nontrivial issues in the detection of causal influences or, at least, non-spurious correlations. Because of the systemic nature of metabolic control, observed correlations are usually small (|ρ| < 0.6) and they cannot be used to measure metabolites distance in a metabolic pathway. Moreover, although the metabolic networks of several organisms like eukaryotes, bacteria or archea, for example, show a power-law degree distribution and a high clustering coefficient [1], metabolite correlation networks have not been fully characterized in terms of network topology [16].

In this paper, we defined and applied a new simple but effective workflow to conduct a weighted differential correlation network analysis on metabolomics data and we propose a classification model based on the inferred network’s information able to measure the explanatory power of the resulting network model and to evaluate if the identified differential associations had a discriminative power. The main novelty of this work relies in the usage of the so-called disparity filter [17] to reduce the network to it is connection backbone. Once the differential network is built, this filter allows the identification of edges relevant with respect to a null model, exploiting the heterogeneity present in the intensity (weights) of the differential links, both at global and local levels, without down-playing small-scale interactions. The advantage of the disparity filter is that the obtained backbone preserves almost all nodes of the initial network and a large fraction of the total weight, while reducing considerably the number of links that pass the filter. Moreover, this procedure does not need any assumption on the distribution of the data.

For the classification task, we chose to adopt the partial least square-discriminant analysis (PLS-DA) method, since it is one of the most popular classification methods in metabolomics. To the best of our knowledge, the application of the disparity filter to differential network analysis and the coupling of it with PLS-DA are original in the metabolomic literature.

We applied our method to a publicly available metabolomic dataset on colorectal cancer (CRC) [18], which includes 224 serum samples from healthy controls and patients who suffer from colorectal cancer or polyp. The proposed network differential analysis allowed to identify hub metabolites and differential association patterns which were consistent with current knowledge and the original paper, and the PLS-DA classifiers were able to distinguish with a good accuracy CRC sample from both healthy and polyp samples, suggesting that the identified differential networks can be meaningful and have discriminative power.

## 2 Architecture and implementation

In metabolomic studies at least two phenotypes are compared, e.g., cases versus controls. The differential network analysis framework here proposed consists of three main steps: the inference of a weighted differential association network (based for the sake of simplicity on Pearson’s correlation), the reduction of such network to its backbone by application of the disparity filter, and the construction of a model that exploits the information obtained from the differential network to classify the two phenotypes.

### 2.1 The weighted differential correlation network

For each pair of metabolites *i* and *j*, we define the differential association measure as follows:

$$\rho_{di\mathrm{ff}}\left(i,j\right)={\left|\rho_{cases}\left(i,j\right)-\rho_{controls}\left(i,j\right)\right|}^{\gamma },\gamma >1,$$

where $\rho_{cases}\left(i,j\right)$ and $\rho_{controls}\left(i,j\right)$ are the Pearson’s correlation coefficients for metabolites *i* and *j* in the two conditions, respectively and the factor *γ* > 1 is chosen to push low differences towards 0, while conserving high values. This differential quantity is a power function of the absolute change of correlation across the two conditions. The statistical significance of each differential association is assessed using a 1000-fold permutation test: briefly, for each permutation, the samples are randomly allocated to one or the other status, in order to remove the relation among metabolites’ correlations and the phenotypes. The statistical significance (p-value) of $\rho_{di\mathrm{ff}}\left(i,j\right)$ is estimated as the proportion of the permuted differential associations that are greater than the observed values calculated using the original real data. Therefore, we define a differential network, where the nodes are metabolites and two metabolites are linked if and only if the *p*-value of their related differential association measure is lower than the fixed cut-off 0.05. Let us note that the so inferred network is naturally weighted on the edges, associating the value of the differential association measure to each correspondent edge.

Despite the limitations due to fact that Pearson’s correlation measure just considers linear interactions, this method allows to infer a differential network using a widely known statistical tool, making the model interpretable and it does not require any conditions on the distribution of the data.

### 2.2 The disparity filter

Given the complex nature of correlation in metabolomics it is necessary to perform a filtering analysis to extract the relevant information from the inferred differential network. Moreover, filtering techniques allow a reduced but more relevant representation while preserving the key differential connections. To this purpose, we propose the usage of the disparity filter presented in [17] that is a network reduction method that exploits the weighted nature of the differential network and it operates at all the scales present in the system. The disparity filter analyses the edges at the node’s level and preserves just the ones that have weights unexpectedly high, or in other terms that represent significant deviations with respect to a null hypothesis of uniform randomness. As a result, this filtering technique significantly reduces the total number of edges, keeping a large fraction of the total weight and unlike the global threshold filter, it preserves the form of the weight distribution, and the clustering coefficient.

The filtering method starts by normalizing the weights *w*_*i*,*j*_ of the *n*_*i*_ edges linked to a certain metabolite *i*, as follows:

$$p_{i,j}=\frac{w_{i,j}}{\overset{n_{i}}{\underset{j=1}{\sum }}w_{i,j}}.$$

The heterogeneity in the local distribution of the edges’ weights insisting on *i* is characterized by the disparity measures [17]:

$$Y\left(n_{i}\right)=n_{i}\overset{n_{i}}{\underset{j=1}{\sum }}p_{i,j}^{2}.$$

This is a function that has been extensively used in complex networks theory and it characterizes local heterogeneity. If all the links have the same weight, we are in a situation of perfect homogeneity and it holds $Y\left(n_{i}\right)=1$ , whereas for perfect heterogeneity, i.e., one of the links carries all the weight, it holds $Y\left(n_{i}\right)=n_{i}$ . In real network, we usually observe intermediate behaviour, proportional to a power function of the node’s degree with exponent close to $\frac{1}{2}$ . As reported in [17], this is the situation when the disparity filter results more useful.

After normalizing the weights, the disparity filter proceeds by identifying which links for each node *i* should be preserved in the network. The null model used for this discrimination is based on the null hypothesis that the *n*_*i*_ normalized weights are produced by a random assignment from a uniform distribution. All the edges that reject the null hypothesis, i.e., those with weights not compatible with the null model, can be considered as significant deviations due to the network-organizing principles. By imposing a cut-off *α*, the relevant edges for a node *i* will be those whose weights satisfy the relation [17]:

$$\alpha_{i,j}={\left(1-p_{i,j}\right)}^{n_{i}-1}<\alpha$$

By lowering the parameter *α*, we can filter out the links progressively focusing on more relevant edges.

The network backbone is therefore obtained by preserving all the edges that satisfy the above criterion for at least one of the two nodes they insist on, while discounting the rest.

### 2.3 The classification model

Once performed the differential network analysis, it is also interesting to study the explanatory power of the proposed differential method and to evaluate if the extracted information can be useful for classification. With this purpose in mind, we define a novel set of features that “translates” the properties of the filtered differential network’s backbone, and it consists of:

– the nodes that are connected to at least another metabolite in the backbone,
– the interaction term, i*j, for each preserved edge in the backbone linking metabolites *i* and *j*.

Thus, this novel set of features can be used to train a classification model. To this purpose, we choose to perform a PLS-DA since it is widely adopted by the metabolomics community and, being a dimensionality-reduction technique, it can handle the intrinsic multicollinearity of the novel dataset. However, other suitable classification methods may be chosen.

## 3 Application

For the evaluation of the proposed method, we used a published metabolic dataset on colorectal cancer (CRC) [18]. The dataset is publicly available at the NIH Common Fund’s National Metabolomics Data Repository (NMDR) website, the Metabolomics Workbench, https://www.metabolomicsworkbench.org, where it has been assigned the Project ID PR000226. The data can be accessed directly via its Project DOI: https://doi.org/10.21228/jib-2021-0030.

Briefly, metabolomics was performed on serum samples of 234 subjects (both healthy and patients) undergoing either colonoscopy or CRC surgery; samples were collected after overnight fasting and bowel preparation. The groups consisted of healthy controls (*n* = 92), CRC patients (*n* = 66), and patients with colorectal polyps (*n* = 76), based on colonoscopy examination results. Patients were age- and gender-matched in each group. A targeted liquid chromatography-tandem mass spectrometry (LC−MS/MS) approach was used for comprehensive CRC serum metabolic profiling under a standard operating procedure. In total, 113 metabolites were reliably detected [18].

In this analysis, after a log-transformation, we randomly split the dataset into training (70%) and test set (30%) with equal balance among the groups and applied the proposed method for differential network analysis to the training set.

For the differential association measure, the power function parameter *γ* was set to 4 to obtain a scale-free distribution of the weights. Moreover, we recall that the disparity filter was applied to a network with statistically significant edges, tested by permutation test. Therefore, in order to reduce considerably the number of links, while maintaining most of the total weight, we set the cut-off *α* of the disparity filter to 0.3. Networks’ topology was then exploited to identify key metabolites in the differential networks. Two centrality measures were considered: nodes’ degree i.e., the number of edges insisting on a node, and betweenness i.e., the number of times a node is part of the shortest path between any pair of nodes [19]. In simple terms, nodes with high degrees are usually referred to as “hubs” since they have more connections than the rest of the nodes in the network, while nodes with high betweenness may be considered as “bottlenecks” since they are crucial in controlling the information flow.

The two differential network analyses were performed on the training set. Then, for each of the comparisons, we considered the novel dataset, enriched with the network backbone information, as explained before, and we trained a PLS-DA model on the training set, aiming to classify subjects according to their phenotype. To avoid overfitting and improve the reproducibility of the results, the classifier was validated through 20-times repeated 10-fold cross validation. The optimal number of dimensions for the PLS-DA model was determined by cross validation. Then, we tested the final model on the remaining part of the subjects (test set). The performance during cross-validation and testing was measured by accuracy and AUROC measure. Variable importance in projection (VIP) measure was adopted to evaluate the variables contribution to the classification models.

The analyses were conducted using the R software version 4.0.5.

## 4 Results

The proposed workflow was applied using a metabolomic dataset previously published that involved patients with CRC versus healthy controls or subjects with colorectal polyps.

### 4.1 CRC versus healthy subjects

**Figure 1:**
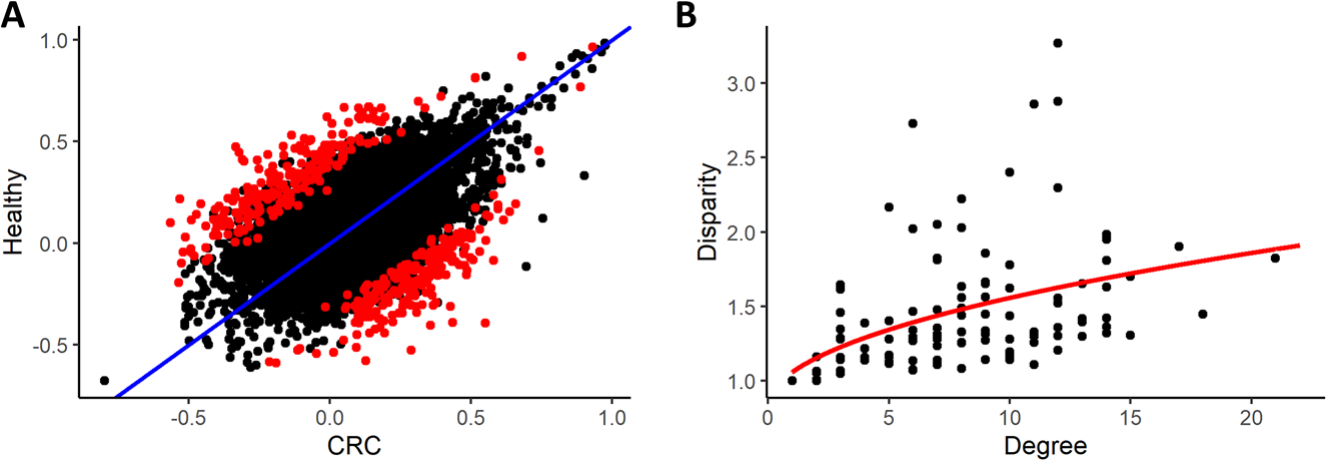
In panel (A), scatterplot of metabolites pair correlations in CRC subjects (*x* axis) and healthy (*y* axis) and identification of significant (*p*-value cutoff 0.05 using a 1000-fold permutation test) pairs in red. In panel (B), scatterplot of the disparity measure (*y* axis) in function of the nodes’ degree (*x* axis) and the fitting curve $y\propto x^{\frac{1}{2}}$ .

The differential network of the statistically significant differential correlations (by permutation test) between CRC and healthy subjects consists of 111 nodes and 450 edges (Figure 1A). The degree’s distribution showed high variability, spanning from 0 to 21 edges per node and average degree 8. At the local level, the heterogeneity of the nodes presented a skewed distribution and, as function of the nodes’ degree, the disparity measure $Y\left(n_{i}\right)$ was found proportional to ${n_{i}}^{\frac{1}{2}}$ (Figure 1B). This means that for most of the nodes the weights insisting on them are peaked on a small number of links and the remaining connections carry just a small fraction of the node’s strength. In this situation, the disparity filter is particularly useful, extracting structures impossible to detect using the more common global threshold filter.

**Figure 2:**
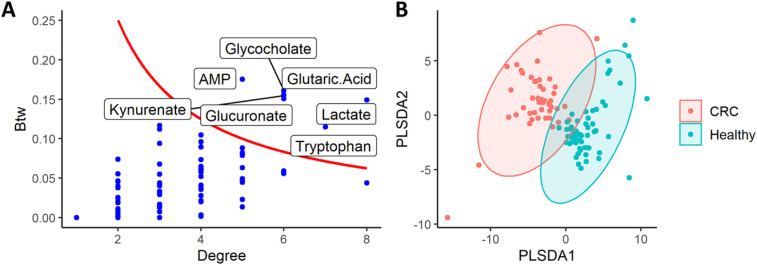
In panel (A), the importance of nodes preserved in the differential network’s backbone between CRC and healthy subjects, characterized by betweenness centrality (Btw, *y*-axis) and node degree (*x*-axis). Key nodes with high degrees and high betweenness (degree*betweenness > 0.5) were labelled with their metabolite names. In panel (B), scores plot of PLS-DA classification model between CRC subjects and healthy controls.

The network’s backbone obtained after applying the disparity filter with a threshold *α* = 0.3 was comprised by one connected component of 100 nodes and 158 edges, preserving just the 35% of the edges but at the same time the 64% of total weight. By analysing the topology of the extracted backbone, we were able to detect 7 central metabolites, which are both hubs and bottlenecks for the differential interactions of the resulting network (Figure 2A). Those metabolites are related to amino acids metabolism, like glutaric acid, kynurenic acid and tryptophan, the energy metabolism (lactate and adenosine monophosphate), plus glucuronic acid and glycocholate. Although these results are in agreement with the published findings, it is worth noting that only two of these metabolites (glycocholate, kynurenic acid) showed a significative difference in the distribution (Mann–Whitney’s test *p*-value < 0.05) and therefore, were considered relevant in the original paper [18, p. 4123]. The other five metabolites, while not significantly different in concentration and therefore not detectable with standard analysis, resulted central in the differential network, suggesting a role of such metabolites in CRC occurrence.

To obtain a predictive model and at the same time to assess the explanatory power of the resulting network model, we trained a network-based PLS-DA for the classification of CRC and healthy subjects, as detailed in the previous section. The classification model was able to distinguish the two groups (Figure 2B), with high accuracy and AUROC (95% and 0.98 from cross-validation; 80% and 0.81 on the independent test set, respectively). The other performance measurements on the testing sub cohort reported in Table 1, like F1 score or positive predictive values, confirmed the goodness of our classification model. Among the features that contribute the most to such classification (VIP > 1.25) there were 12 nodes and 45 interaction terms, suggesting that the differential information extracted with the disparity filter is meaningful and it plays a significant role in the discrimination. As shown in Table 2, the variables with higher discriminative power according to VIP score were also significantly different in distribution (Mann–Whitney’s test *p*-value < 0.05, after FDR adjustment).

**Table 1:** Models’ performance measurements for both classification tasks, CRC versus healthy subjects and CRC versus polyp subjects, respectively.

	Testing cohort	
	CRC vs healthy	CRC vs polyp
Accuracy	0.80	0.80
F1 score	0.78	0.78
AUROC	0.82	0.83
Sensitivity	0.73	0.82
Specificity	0.86	0.79
PPV	0.84	0.74
NPV	0.76	0.86
FNR	0.27	0.18

The F1 score is defined as the harmonic mean of PPV and sensitivity, i.e. 2 × (PPV × sensitivity)/(PPV + sensitivity). AUROC: area under the curve; FNR: false negative rate; PPV: positive predictive value; PVN: negative predictive value.

**Table 2:** Top 25 relevant variable (according to the VIP score) for the PLS-DA model on the enriched dataset between CRC and healthy subjects.

Metabolites	VIP score	Healthy (mean ± SD)	CRC (mean ± SD)	*p*-Value
4-Pyridoxic acid – niacinamide	1.82	−0.25 ± 0.77	0.34 ± 0.92	0.0003
Glycochenodeoxycholate – malonic acid	1.74	0.05 ± 0.87	−0.75 ± 1.03	0.0003
Linolenic acid	1.71	0.21 ± 0.76	−0.57 ± 1.24	0.0053
Histidine	1.65	0.26 ± 1.06	−0.52 ± 0.95	0.0003
*N*2-*N*2 Dimethylguanosine – GSH	1.58	−0.31 ± 1.15	0.33 ± 1.17	0.0283
Glycochenodeoxycholate – linolenic acid	1.56	0.05 ± 0.9	−0.68 ± 1.42	0.0110
Cytidine – shikimic acid	1.53	0.44 ± 1.17	0.06 ± 0.98	0.0576
5-Hydroxytryptophan – guanidinoacetate	1.53	0.3 ± 0.81	−0.31 ± 0.92	0.0038
AMP – glucuronate	1.51	−0.39 ± 1.18	−0.02 ± 0.81	0.0154
Adenosine – kynurenate	1.50	0.21 ± 0.81	−0.3 ± 0.98	0.0145
Glucose – pyridoxal-5-P	1.49	−0.02 ± 0.66	0.25 ± 0.62	0.0505
3-Nitro-tyrosine – dimethylglycine	1.48	−0.24 ± 1.05	0.35 ± 0.84	0.0042
Cystamine – glucose	1.48	0.06 ± 0.74	−0.28 ± 1.02	0.0346
F16BP/F26BP – linolenic acid	1.47	−0.28 ± 0.78	0.35 ± 1.3	0.0083
Glyceraldehyde	1.46	−0.18 ± 0.82	0.59 ± 1.11	0.0003
Kynurenate	1.46	−0.13 ± 0.86	0.29 ± 0.96	0.0281
Urate – uridine	1.44	−0.09 ± 1.14	0.5 ± 1.12	0.0624
d-Glyceraldehyde-3-phosphate (D-GA3P/DHAP) – IMP	1.42	0.21 ± 0.82	−0.19 ± 0.9	0.0576
Urate	1.42	0.12 ± 1.04	−0.28 ± 0.92	0.0535
Glycochenodeoxycholate	1.41	−0.17 ± 1.04	0.47 ± 0.85	0.0014
Homovanilate – taurine	1.40	0.12 ± 0.91	−0.22 ± 0.82	0.0283
Cystamine – lactate	1.40	0.08 ± 0.84	−0.4 ± 1.21	0.0497
d-Glyceraldehyde-3-phosphate (D-GA3P/DHAP) –	1.40	0.34 ± 1.06	−0.33 ± 1.02	0.0057
guanidinoacetate				
4-Pyridoxic acid – glucuronate	1.39	−0.43 ± 1.06	0.16 ± 1.25	0.0018
Inosine – linolenic acid	1.38	−0.21 ± 0.74	0.21 ± 1.03	0.0309

### 4.2 CRC versus polyp subjects

**Figure 3:**
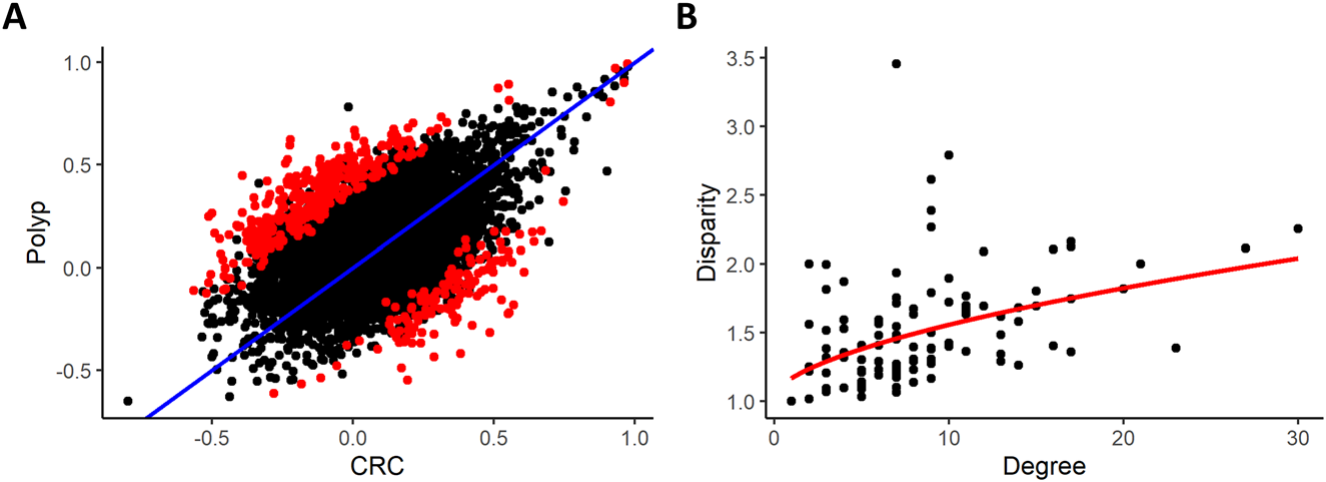
In panel (A), scatterplot of metabolites pair correlations in CRC subjects (*x* axis) and polyp (*y* axis) and identification of significant (*p*-value cutoff 0.05 using a 1000-fold permutation test) pairs in red. In panel (B), scatterplot of the disparity measure (*y* axis) in function of the nodes’ degree (*x* axis) and the fitting curve *y* ∝*x* ^3/5^.

In the comparison between CRC and polyp subjects, we observed a similar performance of the proposed algorithm. The differential network of the statistically significant differential correlations consists of 112 nodes and 462 edges (Figure 3A). The average nodes degree was 7, spanning from 0 to 30 edges linked on one node and, regarding the disparity measure, we assessed that $Y\left(n_{i}\right)\propto n^{\frac{3}{5}}$ (Figure 3B). Therefore, once again we demonstrated the usefulness of the disparity filter in the analysis of this type of data.

**Figure 4:**
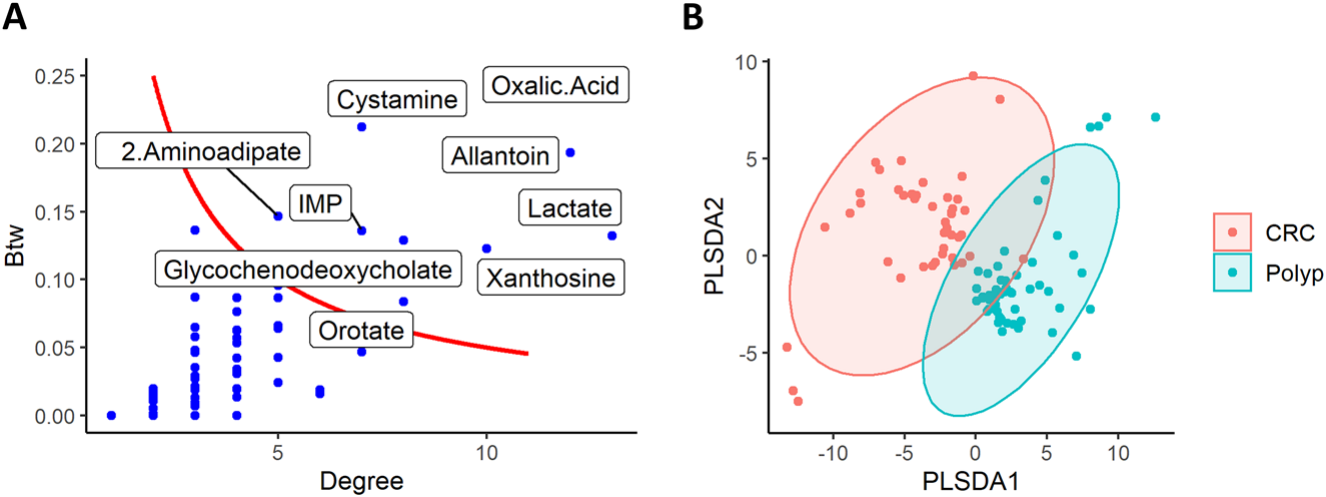
In panel (A), the importance of nodes preserved in the differential network’s backbone between CRC and polyp subjects, characterized by betweenness centrality (Btw, *y*-axis) and node degree (*x*-axis). Key nodes with high degrees and high betweenness (degree*betweenness > 0.5) were labelled with their metabolite names. In panel (B), scores plot of PLS-DA classification model between CRC subjects and polyp controls.

Once applied the disparity filter with threshold *α* equal to 0.3, we filtered out the 64% of the edges, while preserving 65% of total weight. Figure 4A shows the most central metabolites, according to degree and betweenness. Among them, there are metabolites related to energy metabolism (lactate, oxalic acid), amino acids (orotate, 2-aminoadipate), purine metabolism pathway (xanthosine, inosine monophosphate), coherently with the original study [18]. Only two of such central metabolites (glycochenodeoxycholate and orotate) showed a significantly difference in the distribution (Mann–Whitney test’s *p*-value < 0.05), remarking once again that the methodology here proposed offers a complementary view with respect to standard statistical analysis.

The PLS-DA classifier was able to distinguish the two groups with an accuracy of 95% and an AUROC of 0.97 from cross-validation. Despite the slight decrease in performance we expected, the classification models obtained good results on the independent testing set, too (accuracy 80%, AUROC 83%; see Table 1). Among the top 25 features that contribute the most to such classification according to VIP score (Table 3), there were 6 nodes and 19 edges, most of them significantly different between the two groups (Mann–Whitney’s *p*-value < 0.05, FDR adjusted), confirming that detected relevant interaction terms play a role in the discrimination, also in this case.

**Table 3:** Top 25 relevant variable (according to the VIP score) for the PLS-DA model on the enriched dataset between CRC and polyp subjects.

Metabolites	VIP score	Polyp (mean ± SD)	CRC (mean ± SD)	*p*-Value
Cytidine – trimethylamine-*N*-oxide	1.80	0.44 ± 1.51	−0.13 ± 0.99	0.01
Lysine	1.77	0.31 ± 0.88	−0.48 ± 1.01	0.01
Methionine	1.70	0.28 ± 0.89	−0.46 ± 0.96	0.01
Linolenic acid	1.65	0.24 ± 0.8	−0.57 ± 1.24	0.02
Glyceraldehyde	1.64	−0.3 ± 0.87	0.59 ± 1.11	0.01
2′-Deoxyuridine	1.62	0.24 ± 0.91	−0.45 ± 0.97	0.02
Cystamine – glucose	1.61	0.08 ± 0.73	−0.28 ± 1.02	0.02
Cytidine – glycochenodeoxycholate	1.61	0.31 ± 0.84	−0.12 ± 0.78	0.02
Adenosine – glycochenodeoxycholate	1.58	0.22 ± 0.87	−0.36 ± 0.78	0.01
Allantoin – PEP	1.57	0.55 ± 1.2	−0.14 ± 0.65	0.01
4-Pyridoxic acid – glycine	1.51	−0.12 ± 1.06	0.54 ± 1.5	0.02
Glycochenodeoxycholate	1.51	−0.21 ± 0.95	0.47 ± 0.85	0.02
Cystamine – lactate	1.49	0.04 ± 0.75	−0.4 ± 1.21	0.04
*N*-Acetylglycine – propionate	1.47	0.15 ± 1.05	−0.31 ± 1.14	0.02
d-Glyceraldehyde-3-phosphate (D-GA3P/DHAP) – glycine	1.46	−0.03 ± 0.9	0.52 ± 1.1	0.04
Allantoin – GSH	1.44	−0.61 ± 1.19	0.03 ± 0.8	0.02
Glucose – hydroxyproline/aminolevulinate	1.44	0.18 ± 0.79	−0.21 ± 0.89	0.03
gamma-Aminobutyrate – *N*-acetylglycine	1.43	−0.06 ± 0.86	0.62 ± 1.42	0.04
l-Kynurenine – GSH	1.42	−0.16 ± 0.73	0.32 ± 1.15	0.01
Fructose – PEP	1.42	0.49 ± 0.84	0.001 ± 0.92	0.03
Cystamine – oxalic acid	1.42	0.01 ± 0.78	−0.37 ± 1.16	0.03
Hydroxyproline/aminolevulinate – lactate	1.41	0.21 ± 0.81	−0.3 ± 1.29	0.07
Allantoin – methylsuccinate	1.41	0.24 ± 1.15	−0.19 ± 0.95	0.05
Glycochenodeoxycholate – sorbitol	1.39	−0.18 ± 0.82	0.37 ± 1.21	0.02
Allantoin – asparagine	1.38	0.19 ± 1.22	−0.2 ± 0.93	0.08

## 5 Conclusions

A complex disease phenotype, like cancer, alters different biological mechanisms that interact in a network [1]. Here, we developed a simple but effective framework to perform differential network analysis and applied it to a published CRC metabolomics dataset. Focusing on differential interactions rather than differential concentrations, network differential analysis offers a complementary perspective with respect to standard analysis techniques and it has become an important tool for the analysis of the underlying pathophysiological processes. The evaluation of the CRC dataset using differential network analysis showed that the method here proposed provided useful insights into the backbone of the differential interactions between two phenotypes (presence or not of cancer) and was able to achieve classification. Compared to the original analyses, this methodology revealed important alterations of the interactions network occurring in CRC with respect to both healthy and polyp subjects and it allowed the identification of several novel metabolites which resulted central in the differential information flow, although further validations will be necessary.

In conclusion, the proposed method is an easy-to-use novel approach for reconstruction and analysis of differential association networks and may constitute a first step towards inferring causal relationships and discovering novel candidate biomarkers.
